# A Convolutional Autoencoder Based Fault Diagnosis Method for a Hydraulic Solenoid Valve Considering Unknown Faults

**DOI:** 10.3390/s23167249

**Published:** 2023-08-18

**Authors:** Seungjin Yoo, Joon Ha Jung, Jai-Kyung Lee, Sang Woo Shin, Dal Sik Jang

**Affiliations:** 1 Department of Smart Industrial Machine Technologies, Korea Institute of Machinery and Materials, Daejeon 34103, Republic of Korea; jkleece@kimm.re.kr; 2 Department of Industrial Engineering, Ajou University, Suwon 16499, Republic of Korea; joonha@ajou.ac.kr; 3 R&D Center, Daesung Nachi Hydraulics Co., Ltd., Yangsan 50592, Republic of Korea; ssw87@daesung.co.kr (S.W.S.); kairosjang@daesung.co.kr (D.S.J.)

**Keywords:** fault diagnosis, solenoid valve, convolutional autoencoder, unknown class classification

## Abstract

The hydraulic solenoid valve is an essential electromechanical component used in various industries to control the flow rate, pressure, and direction of hydraulic fluid. However, these valves can fail due to factors like electrical issues, mechanical wear, contamination, seal failure, or improper assembly; these failures can lead to system downtime and safety risks. To address hydraulic solenoid valve failure, and its related impacts, this study aimed to develop a nondestructive diagnostic technology for rapid and accurate diagnosis of valve failures. The proposed approach is based on a data-driven model that uses voltage and current signals measured from normal and faulty valve samples. The algorithm utilizes a convolutional autoencoder and hypersphere-based clustering of the latent variables. This clustering approach helps to identify patterns and categorize the samples into distinct groups, normal and faulty. By clustering the data into groups of hyperspheres, the algorithm identifies the specific fault type, including both known and potentially new fault types. The proposed diagnostic model successfully achieved an accuracy rate of 98% in classifying the measurement data, which were augmented with white noise across seven distinct fault modes. This high accuracy demonstrates the effectiveness of the proposed diagnosis method for accurate and prompt identification of faults present in actual hydraulic solenoid valves.

## 1. Introduction

A hydraulic system is a self-contained system that produces large pressure from a relatively small-sized device. Thus, hydraulic systems are used in various applications such as automotive systems, construction machinery, agriculture equipment, and in many other industrial settings [1]. All of these hydraulic systems have a valve that controls the pressure of the fluid; an exact amount of pressure can be supplied to each required part. One valve type that is frequently used is the hydraulic solenoid valve (HSV), which is an electromechanical device.

The principle of an HSV is to control a valve using a supplied electrical current [2]. Specifically, when an electrical current is applied to the solenoid coil, the coil generates a magnetic field that pulls or pushes the plunger (or spool), depending on the design of the valve. This movement of the plunger opens or closes the fluid ports, controlling the flow of the fluid. Thus, HSVs have the following features: fast response times, accurate control, and the ability to operate remotely or automatically. In addition, automatic logic can be embedded into a low-powered microprocessor for use in HSVs. Overall, HSVs play a significant role in hydraulic systems by providing reliable and precise control of fluid flow. Sensitive control of the fluid increases the efficiency and the functionality of various mechanical and industrial processes.

However, like any mechanical device, HSVs can fail due to various causes such as coil failure, contamination, mechanical wear, seal degradation, and/or improper assembly. For example, if a solenoid coil is overheated, insulation of the coil may break down, and the valve may not receive the electrical control current, leading to a failure in opening or closing the valve. In addition, hydraulic systems are susceptible to contamination by dirt, debris, or foreign particles. If these contaminants go into the solenoid valve, the contaminants can hinder the movement of the plunger or spool, causing the valve to malfunction. Further, the components of the solenoid valve, such as the plunger, spool, or valve seat, can experience wear and performance degradation. A component with worn parts may lead to leakage, improper sealing, or reduced responsiveness of the valve. Physical damage to the solenoid valve, such as from impact or improper handling, can cause misalignment, bending, or even fracture of the internal components. Furthermore, failures can occur due to electrical issues as well. For example, improper wiring can prevent the valve from receiving accurate electrical signals, resulting in failure.

The failure of an HSV lowers the efficiency of the hydraulic system because the pressure cannot be controlled. Moreover, with some failures, the entire hydraulic system can stop, which leads to downtime and economic loss. Further, in a large system, uncontrollable pressure may even cause casualties. Therefore, to prevent HSV failures, there is an increasing need to monitor the health state of HSVs and implement a fault–diagnosis function that accurately classifies its health state. An accurate diagnosis system for an HSV can identify and address issues early, improving reliability, functionality, and extending the life of the hydraulic system. As a result, failures and downtime of an HSV can be reduced, which saves operation and maintenance costs.

Thus, research has increasingly been conducted to develop such a diagnosis system for HSVs. Conventional methods for diagnosing hydraulic solenoid valves typically involve a combination of visual inspection, functional testing, and measurement techniques. Visual inspection is one basic diagnosis method. This method checks an HSV visually to look for any visible contaminants or blockages that may be affecting the valve’s operation. Physical damage, such as cracks, leaks, loose connections, or bent parts, can be found in a visual inspection. Another method is function testing of the solenoid valve. Functional testing is performed by connecting the valve in a hydraulic circuit and testing the flow of hydraulic fluid through the valve. During the test, the flow rate and pressure is measured and matched to the designed specifications. Any abnormal pressure drops or irregularities in the flow indicate an abnormal status of the valve.

However, these conventional methods are not preferred for the in-line diagnosis of HSVs and for the application where the health state of the part should be checked frequently and rapidly. The visual inspection requires a great amount of human effort and time, which is costly and can involve human error. Likewise, functional testing requires separate equipment to test the performance of an HSV, which requires disassembly of the hydraulic system, which is a destructive testing method.

Therefore, there has been research for the in-line diagnosis of HSVs, which can perform nondestructive and rapid diagnosis without disassembly. Data-driven fault diagnosis for HSVs has been explored by several different researchers in recent years. In [3], a nonintrusive fault-diagnosis method was presented for a solenoid valve, using a multifeature fusion approach. The method combines time–domain and time–frequency–domain analysis of the current signal. In [4], a fault diagnosis method based on multikernel support vector machine (MKSVM) is introduced. The proposed method was validated using four different real fault samples of hydraulic solenoid valves. In [5], an optimal sensor arrangement method is presented for fault diagnosis of hydraulic control system. The method determines the optimal number and position of sensors based on a discrete particle swarm algorithm. In addition, other research has attempted to diagnose solenoid valves using proposed hand-crafted features with classification algorithms [6,7].

Despite some successful results in these previous studies, there are still some constraints that need to be considered. First, the previous studies relied on data acquired from an extra sensor attached to the device [8,9]. Although an extra sensor can acquire consistent signals, placing an extra sensor may not be possible to certain hydraulic systems. In addition, the hand-crafted features used in the prior studies rely heavily on the knowledge of experts, which may not be available in all settings.

Recent studies have tackled these aforementioned issues through the use of control signals and the deep learning approach. In [10,11,12,13], methods for estimating the displacement of hydraulic solenoid valves are discussed. Since the inductance changes according to the plunger displacement in a solenoid valve, the displacement of the plunger is estimated by analyzing the current and voltage signals of the valve. In [14,15], the flux linkage induced in the coil of a hydraulic solenoid valve is estimated based on the current and voltage signals. The study presents two-dimensional plots of the current and flux linkage for different types of valve faults, and articulated the usefulness of the curve without an extra sensor. In [16,17], the authors developed a one-dimensional convolutional neural network (CNN) model to classify valve faults. By analyzing the current–flux linkage curve of the valve, the model was able to accurately detect faults and categorizes them into predefined fault modes. This approach demonstrates the potential effectiveness of using CNNs for fault detection and classification in solenoid valves.

In spite of the recent studies showing outstanding results, certain limitations still exist. One significant drawback is that these studies have been confined to known faults, disregarding the possibility of unknown faults occurring during operations. The classification capabilities of the previously proposed methods are limited to the classes used in the training process. Since the acquisition of every possible fault state is not realistic, these conventional methods can be used in a limited condition. Besides, conventional studies have considered two to three fault states, whereas reliable diagnosis system must consider more diverse fault types.

To address these challenges, this paper proposes an innovative method for fault classification in hydraulic solenoid valves that accounts for the presence of unknown health states. The proposed diagnosis approach aims to develop a nondestructive method to facilitate accurate and fast diagnosis of failures in hydraulic solenoid valves. This research uses measured data, including the voltage and current, from both normal and faulty samples of the solenoid valve. To enhance performance, data-processing steps including resampling and augmentation are introduced. These processed data are then fed into a convolutional autoencoder, enabling automatic feature extraction. Subsequently, fine-tuning of the autoencoder during the classification process is used to further enhance the prediction accuracy of the hydraulic solenoid valves (HSV) diagnosis approach. Overall, the evaluation of this diagnostic model shows a minimum per-class accuracy rate of 98% across seven distinct modes.

The remainder of this paper proceeds as follows: Section 2 provides an overview of hydraulic solenoid valves and the test procedure. Section 3 outlines the proposed HSV diagnosis method. Section 4 provides details about the experimental setup, and Section 5 presents the results obtained from the proposed method using the experimental data. Finally, Section 6 presents the conclusions of this research, underscoring the significance of the proposed approach in advancing fault diagnosis for hydraulic solenoid valves.

## 2. Overview of Hydraulic Solenoid Valves and the Test Procedure

### 2.1. Hydraulic Solenoid Valve

**Figure 1:**
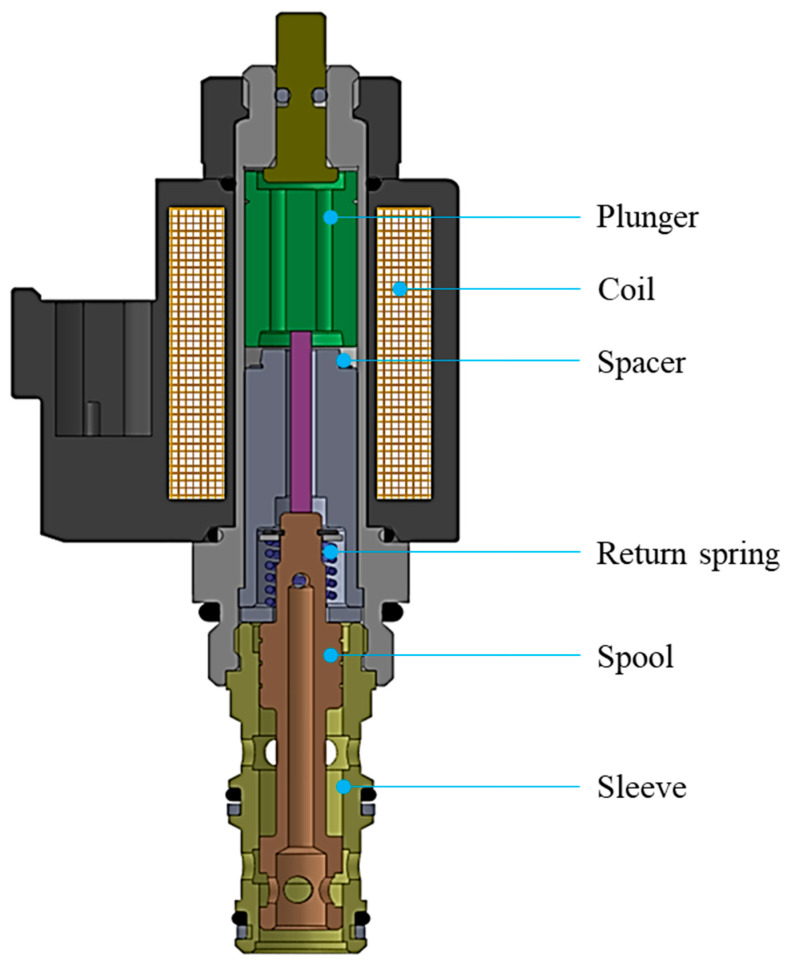
Schematic view of a hydraulic solenoid valve.

This research focuses on developing a fault-diagnosis method for hydraulic solenoid valves (HSVs) that are of the electronic proportional pressure reduction (EPPR) type, which is one of the frequently used HSVs. Figure 1 presents a cross-sectional view of an EPPR valve. EPPR valves control the outlet pressure of hydraulic fluid with respect to the input current. When current flows through the coil, the plunger and the spool move, controlling the pressure through electromagnetic force. In this process, the magnitude of the generated electromagnetic force is proportional to the magnitude of the current.

The EPPRV used in this research can control the pressure of the fluid up to 25 bar. To control the fluid at the maximum pressure, the maximum current of 700 mA is supplied. In addition, the coil driving voltage uses 24 volts, which is driven through a pulse-width modulation (PWM) control with a carrier frequency of 120 Hz.

### 2.2. Test Procedures

The health state of an electromechanical device can be presented through the flux linkage. Since the flux linkage curve shows patterns according to the health state of the device, the flux linkage curve is frequently used to test the electromechanical device [14,15]. Thus, this section describes the nondestructive testing procedure to acquire the flux linkage curve from an HSV.

**Figure 2:**
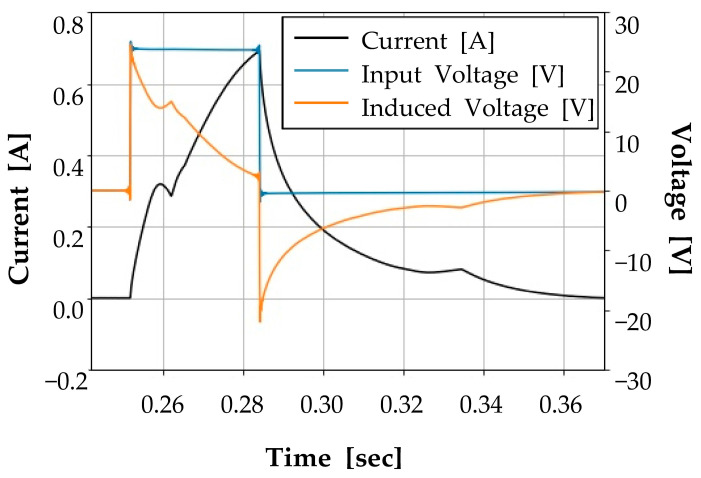
Current and voltage signals during the test procedure.

The test procedures are presented in Figure 2. The first step of the test is to turn on the power until the current reaches its maximum value. Then, the power is turned off when the current reaches its maximum value. The current and the input voltage (voltage between the coil terminals) are continuously measured until the current signal drops to zero. While the current signal decreases slowly from its maximum value, the induced voltage will sharply drop to a negative value and slowly converge to a zero value. The negative input voltage occurs when the current flows through the freewheeling diode right after the power is cut off. At this moment, the voltage drop caused by the diode produces negative voltage at the coil terminals.

## 3. Autoencoder Based Fault Diagnosis for a Hydraulic Solenoid Valve

**Figure 3:**
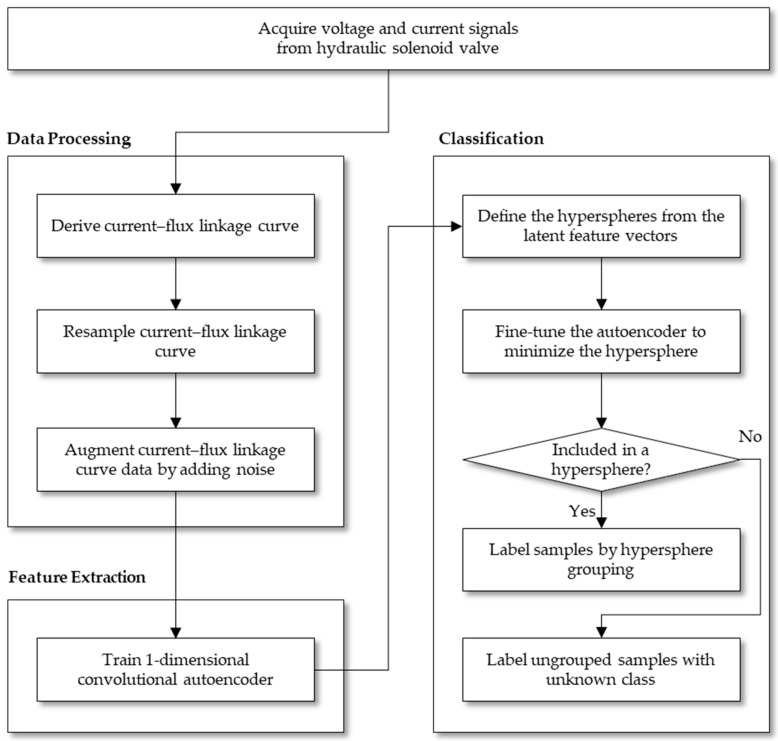
Flow chart of the proposed fault diagnosis method for hydraulic solenoid valves.

This section describes the proposed fault diagnosis method for a solenoid valve using voltage and current signals. The overall procedure is displayed in Figure 3. The voltage and current signals of a hydraulic solenoid valve shows different patterns according to the health state of the valves. Specifically, a flux linkage drawn with voltage and current signals shows different shapes when the valve is turned on and off. Thus, the proposed method uses the flux linkage data to accurately classify the health state of the valves. By converting the flux linkage data into vector data, feature vectors are then extracted from the latent variables of a one-dimensional autoencoder. Finally, the feature vectors are clustered with hyperspheres, and the validated samples are labeled according to the hyperspheres. However, if a sample is not included in any of the hyperspheres, then the sample is labeled as an unknown class or an unknown fault. A detailed description of the proposed method is provided in the following subsections.

Section 3.1 describes the detailed data processing steps that convert voltage and current signals into a flux linkage vector data. In Section 3.2, the feature extraction module of the proposed method is outlined, and in Section 3.3, the classification of the latent variables based on the hypersphere is explained.

### 3.1. Data Processing

The proposed method uses the current–flux linkage curve to diagnose different faults of a hydraulic solenoid valve. The flux linkage (Ψ), also known as magnetic flux, is a function of the current, and the flux data can be obtained through a nondestructive testing process, as described in Section 2.2. The magnetic flux cannot be measured through a sensor, but it can be derived from the measured input voltage and the current.

The following subsections will describe the detailed process of converting the current–flux linkage curve to an input vector for the one-dimensional autoencoder.

#### 3.1.1. Derivation of the Current–Flux Linkage Curve

Equation (1) presents the voltage equation of a solenoid coil [18,19,20].

(1) $$\frac{d\Psi }{dt}+iR=V$$

Ψ presents a coil flux linkage, t is time, i presents the current, R signifies the resistance of the coil, and V indicates the voltage of the coil. Note that the flux linkage (Ψ) of a coil is a function of the current (i) and the plunger displacement (x). By integrating Equation (1) with respect to time, t, the flux linkage can be written as shown in Equation (2).

(2) $$\Psi \left(t\right)-\Psi \left(0\right)=\int_{0}^{t}V-iR\;dt$$

The resistance of coil, R, can be assumed to be a constant during the testing period [0,T], then R can be calculated as shown in Equation (3).

(3) $$R=\frac{\Psi \left(0\right)-\Psi \left(T\right)+\int_{0}^{T}V\;dt}{\int_{0}^{T}i\;dt}$$

T denotes the time duration from when power is applied to the coil until the current decreases to zero.

**Figure 4:**
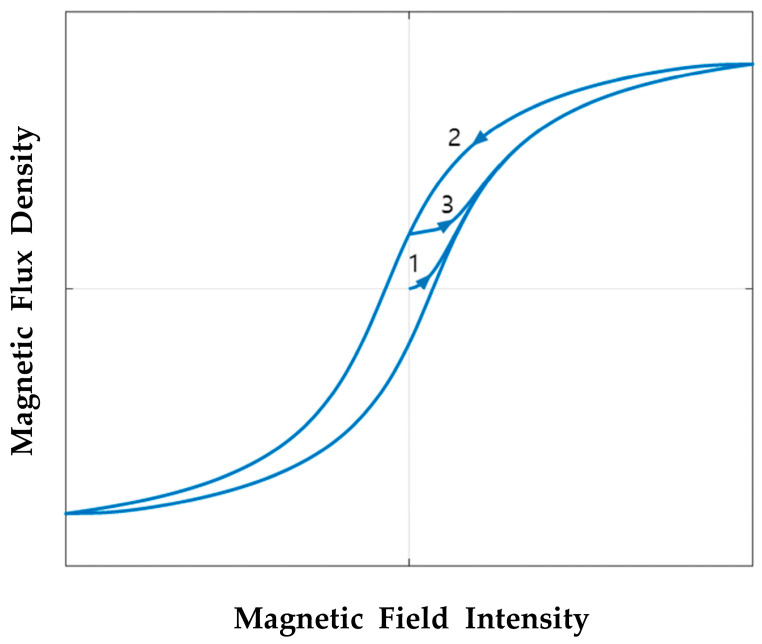
Magnetic hysteresis of a solenoid valve: curve 1 indicates when magnetic field is applied; curve 2 indicates when magnetic field is removed; curve 3 indicates when magnetic field is applied again.

However, due to the magnetic hysteresis phenomenon, the flux linkage at time zero and time T can be assumed to be identical, which means $\Psi \left(T\right)=\Psi \left(0\right)$. The magnetic hysteresis phenomenon is shown in Figure 4. When a magnetic field is applied to the unmagnetized material, the material will follow curve 1 and become magnetized. When the magnetic field is removed, the magnetic flux of the material will decrease but not to zero. The material will retain residual magnetization, shown as curve 2, which is called magnetic hysteresis. If the magnetic field is applied again, the magnetic flux will follow curve 3, and when the field is removed, the material will be demagnetized and appear as curve 2.

As all the tests in this research were performed after the power was switched on and off at least two times, the assumption $\Psi \left(T\right)=\Psi \left(0\right)$ can be applied to Equation (3). Thus, the resistance of the coil can be calculated using the current and the voltage as described in Equation (4).

(4) $$R=\frac{\int_{0}^{T}V\;dt}{\int_{0}^{T}i\;dt}$$

**Figure 5:**
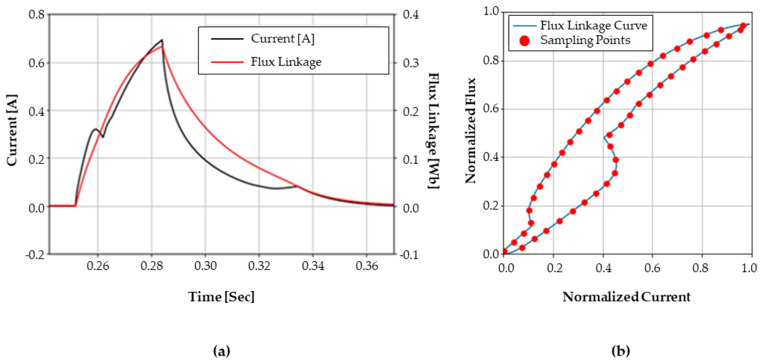
An example of a normalized current−flux linkage curve: (**a**) current and flux linkage trend with respect to time and (**b**) two-dimensional plot between current and flux linkage.

As a result, the current–flux linkage curve during the test period presents a closed-curve shape. To achieve better convergence for training the autoencoder, both the flux linkage and the current are normalized as stated in Equations (5) and (6), respectively. An example of a normalized current–flux linkage curve is shown in Figure 5.

(5) $$\hat{\Psi }\left(t\right)=\frac{\Psi \left(t\right)-\Psi \left(0\right)}{\Psi_{max}}$$

(6) $$\hat{i}\left(t\right)=\frac{i\left(t\right)}{i_{max}}$$

#### 3.1.2. Resampling of Current–Flux Linkage Curve

The normalized current–flux linkage curve presents different shapes according to each health state. Although the curve in Figure 5 is presented as a continuous line, the curve is actually a set of vector data. A curve is derived from a test that takes about 0.15 s, which is equivalent to 1500 samples, if the sampling frequency is set to 10 kHz. Although all 1500 samples can be used to represent the shape of the curve, we suggest a resampling method to reduce the number of samples from 1500 to 50. If the data are to be resampled based on the same time interval, the samples on the curve will not be evenly distributed since the current–flux presents non-linear characteristics, as shown in Figure 5a.

The proposed resampling process consists of two steps. First, the point of the normalized current–flux linkage curve, which is sampled at 10 kHz, is stored whenever the distance of the point from the previously stored data is of $2\sqrt{2}/100$. Considering that the current–flux linkage diagram is a closed curve, and the diagonal length of the normalization plane is $\sqrt{2}$, about 100 points of data can be sampled through this process. However, the length of the data sampled in this way varies depending on the shape of the current–flux linkage diagram. Thus, to make the number of samples consistent, resampling of 48 samples was performed for the next step by interpolating the sampled data in the previous step. Consequently, the original curve with approximately one thousand five hundred-by-two sample data was reduced to forty-eight-by-two curve vector data. The curve vector data is derived from every test and is used as the input of the feature extractor.

#### 3.1.3. Augmentation of the Current–Flux Linkage Curve

The vector data of the current–flux linkage curve is augmented with white noise to consider various operating conditions, which can affect the performance of a diagnosis method. Although the testing can be performed repeatedly, the variation that occurs in actual operating conditions is rarely considered. Consequently, to increase the generalized performance of the feature extractor, a large number of high-quality data is required. However, testing of faulty samples requires a large amount of time and effort, which limits the number of test cases.

Thus, augmentation is performed to increase the size of the data. Specifically, for each flux point, a noise of ±2.5% at each sample point is added, independently. Thus, single curve can be augmented into an infinite number of curves. In this research, the single-curve data are augmented to 1000 different curves.

### 3.2. Autoencoder-Based Feature Learning

In this study, we propose an autoencoder based feature extractor that uses normalized current–flux linkage curve data. Generally, autoencoders are designed with an encoder and a decoder. The encoder network maps the input data into latent features, while the decoder network reconstructs the input from the latent features [21,22,23]. Thus, parameters of an autoencoder are trained by minimizing the difference between the input and the output of the decoder without using label information.

**Figure 6:**
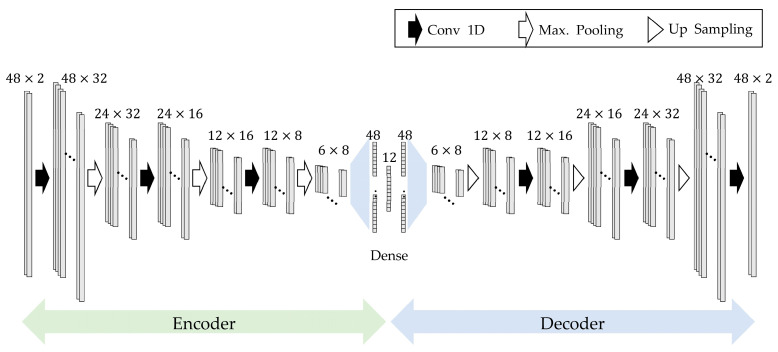
Architecture of the one-dimensional convolutional autoencoder.

As stated in the Section 3.1, the size of the normalized linkage curve data is forty-eight-by-two. As the input vector data include physical characteristics in a temporal dimension, we selected the convolution-based autoencoder. The one-dimensional autoencoder consists of three convolutional layers in the encoder and another three convolutional layers in the decoder. The size of the latent variable between the encoder and the decoder was set to twelve. The overall architecture of the autoencoder is presented in Figure 6. Note that after each convolutional layer, a maximum pooling layer of size two was performed. Also, as in references [24,25], the bias term in the convolutional kernel was removed to increase the training convergence.

In the training process, a loss function of the sum of the mean squared error was used, which is defined as shown in Equation (7).

(7) $$\underset{W}{\min}\sum_{i}{\left\|x_{i}-{\hat{x}}_{i}\right\|}^{2}$$

W denotes parameters of the autoencoder, $x_{i}$ denotes the input vector data of the $i^{th}$ sample, and ${\hat{x}}_{i}$ denotes the output vector data of the autoencoder of the $i^{th}$ sample. The loss function facilitates the network to be trained to predict the output to be as close to the input as possible. When the network is trained as intended, latent variables in the middle of the network can be used as a feature vector. However, the network requires fine-tuning in the classification process, as outlined stated in Section 3.3.

### 3.3. Hypersphere-Based Classification

The classification of a hydraulic solenoid valve (HSV) uses the latent feature vector of the autoencoder, as described in Section 3.2. As the structure of the autoencoder is designed to extract latent features of the current–flux linkage curve data, the extracted latent feature vector can be used for the classification of the HSV health state. In other words, the distribution of the latent feature vector will be separable with respect to each health state.

However, the HSV may encounter an unknown fault mode because labeled data for all possible fault modes cannot be acquired. Thus, an untrained class (or health state) in the classification process is considered in this study; conventional classification methods have not yet explored this issue.

**Figure 7:**
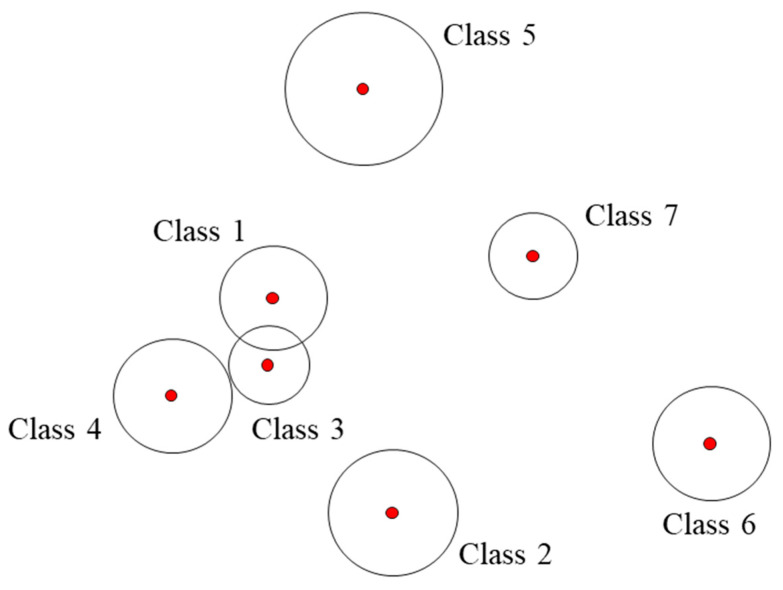
Classification based on hyperspheres.

The proposed hypersphere-based classification scheme is similar to the clustering method, as shown in Figure 7. A hypersphere, which is formed by a latent feature vector, presents each health state, as a latent feature vector of the same health state with a similar distribution. The hyperspheres will be formulated during the training stage. In the validation stage, the label of the sample is predicted based on the result in which the hypersphere region is located. If the validation sample lies within a specific hypersphere region, the sample can be classified as being in the corresponding health state of that hypersphere. However, if the sample comes from an unknown class, the latent feature vector of the sample may be located outside the hypersphere region. Therefore, the sample is classified as a nontrained health state (class), which is an unknown class.

The performance of the hypersphere-based classification scheme relies on the boundary of the region. Thus, we propose a method to define the hypersphere region. First, the data for each health state are grouped into hyperspheres by setting the center point and radius range of the hyperspheres. The center of the hypersphere, $c_{k}$, is defined as the average of the latent feature vectors of the corresponding class k. The following Equation (8) defines the center of the hypersphere:

(8) $$c_{k}=\frac{1}{N}\sum_{i=1}^{N}\phi (x_{k}^{i})$$

where $x_{k}^{i}$ denotes the current–flux linkage curve data of class k, and $\phi \left(\cdot \right)$ denotes the function that maps the current–flux linkage curve data to a latent feature vector in the trained autoencoder. Then, the radius, $R_{k}$, of the hypersphere is determined to include all of the data that belong to class k. Originally, the radius is defined as the maximum distance between the samples and the center of the corresponding class. A sample that is close to the maximum radius can be considered as the same class. Therefore, a margin of ϵ was introduced to consider samples for which the distance to the center is slightly larger than the maximum distance as defined in Equation (9). In this paper, *ε* was set to 0.1.

(9) $$R_{k}=\left(1+\varepsilon \right)\cdot \underset{i}{\max}\left\|c_{k}-\phi \left(x_{k}^{i}\right)\right\|$$

Although a large radius will increase the probability of including all data of each class, it will also increase the likelihood of overlapping regions between hyperspheres, which decreases the classification performance. Thus, to limit the overlapping regions, the autoencoder was fine-tuned to reduce the distance between the samples and the corresponding hypersphere center. The loss term, as defined in Equation (10), was used in the fine-tuning stage of the autoencoder.

(10) $$L_{fine}=\underset{w}{\min}\sum_{k}\sum_{i}{\left\|c_{k}-\phi^{\prime }\left(x_{k}^{i}\right)\right\|}^{2}$$

Here, w denotes the weight parameters of the autoencoder, and $\phi^{\prime }$ denotes the modified mapping function. By fine-tuning the autoencoder with the loss term defined in Equation (10), latent feature vectors are more densely clustered to the center for each class. Finally, the hypersphere is defined using the center $c_{k}$ and the radius $R_{k}$, which is presented as $O(c_{k},\;R_{k})$ throughout this paper.

Even though the autoencoder was fine-tuned, a sample point may locate in the region where hyperspheres are overlapped. Thus, a final prediction is made by the following procedure. If the $i^{th}$ latent feature vector is included in any of the hyperspheres, the sample is classified as the fault mode corresponding to the hypersphere with the closest center; this is described as Condition 1 below. However, if the feature vector is not included in any of the hyperspheres, the sample is classified as an unknown fault; this is described as Condition 2 below.

- Condition 1: If $\phi^{\prime }\left(x_{i}\right)\in O\left(c_{k},R_{k}\right)\;for\;some\;k$, $x_{i}$ is labeled as class k, where $k=\mathrm{argmin}\left\|\phi^{\prime }(x_{i})-c_{k}\right\|$.
- Condition 2: If $\phi^{\prime }\left(x_{i}\right)\notin O\left(c_{k},R_{k}\right)\;\forall \;k$, $x_{i}$ is an unknown fault.

## 4. Description of Datasets

**Figure 8:**
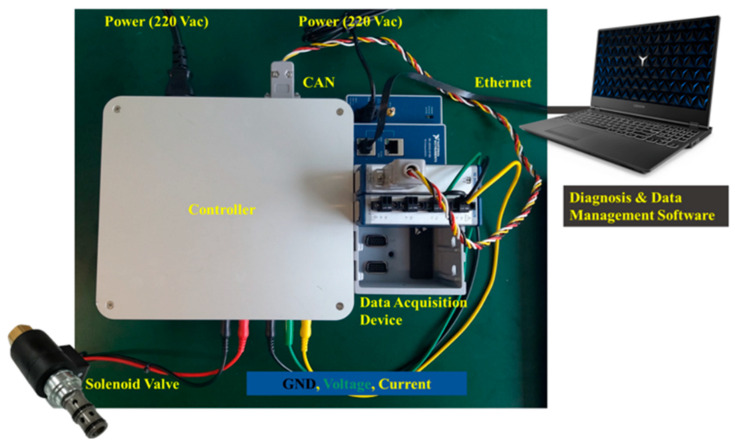
Experimental setup and data acquisition process of the hydraulic solenoid valve test.

**Figure 9:**
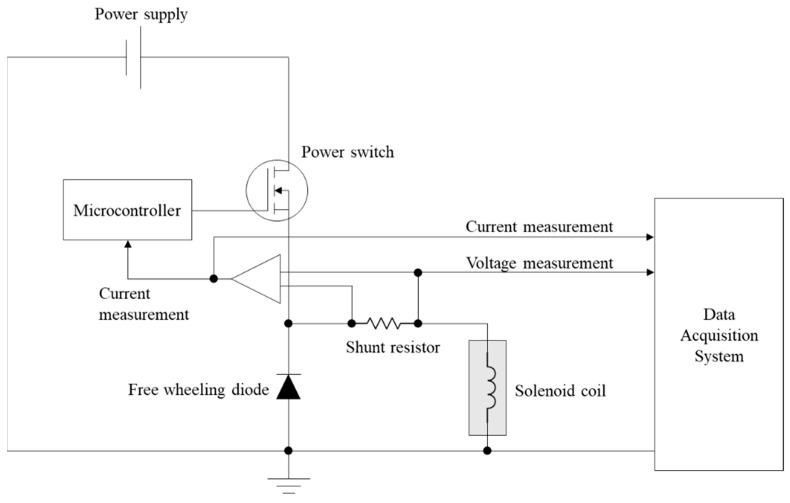
Schematic of the designed controller.

To acquire hydraulic solenoid valve (HSV) data, an experiment was set up as shown in Figure 8. An internal diagram of the controller is shown in Figure 9. A controller was made and attached to the solenoid valve. The controller is controlled through a controller area network (CAN), which is connected via an NI-9862. The output voltage and current from the HSV is acquired through a controller, and the results are converted to digital data via an NI-9229. The two NI devices are connected through a cDAQ-9185. Software to control the valve and acquire data was developed and installed on a host laptop. Note that the data acquisition range was set from −60 V to +60 V with 24-bit resolution, since both the positive and negative voltage comes out from the test as described in Section 2. The sampling frequency was set to 10 kHz.

**Figure 10:**
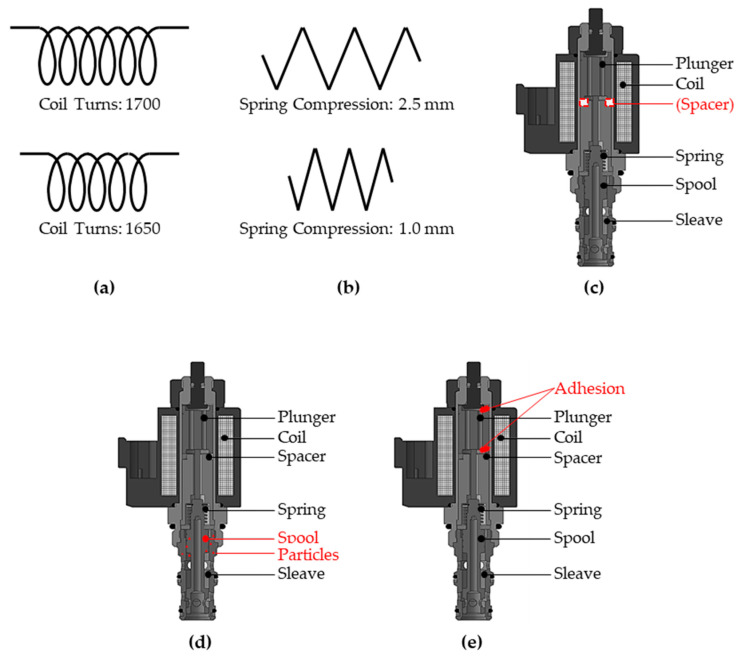
Schematic drawings of test specimens: (**a**) abnormal coil turn, (**b**) abnormal spring stiffness, (**c**) spacer missing, (**d**) spool stuck, and (**e**) plunger adhesion.

Using the testbed, different fault modes of valves were tested. In this study, a normal sample valve and six different fault modes were tested, as shown in Figure 10. A normal valve has 1700 coil turns and 0.06 kgf/mm of spring stiffness. A valve with abnormal coil turns was generated with 1650 coil turns with a larger diameter. Spring stiffness of 0.07 kgf/mm was fabricated by improperly assembling the valve. A spacer was removed to induce a “spacer missing” fault. Foreign substances are applied to the surface of the spool to show the “spool stuck” fault mode. Finally, an adhesion was attached on the top and the bottom of the valve, respectively, for the two fault modes. A total of seven types of valves were tested as summarized in Table 1.

## 5. Results and Discussion

This section describes the results of the proposed method. Section 5.1 describes the result of the data processing, and Section 5.2 outlines the features extracted by the convolutional autoencoder. In Section 5.3, the classification results of the fine-tuned process are detailed. Section 5.4 provides an analysis of the classification results obtained by the hypersphere clusters.

### 5.1. Results of Data Processing

The data processing step includes the derivation of current–flux linkage curve data, resampling, and augmentation. To derive the current–flux linkage curve, the flux linkage needs to be calculated from the measured input voltage and current. The induced voltage is a physical quantity that represents the rate of change of the flux linkage (dΨ/dt), which is stated in Equation (1), the flux linkage can be calculated from the numerical integration of the induced voltage. Also, the resistance is calculated using Equation (4) to derive the flux.

**Figure 11:**
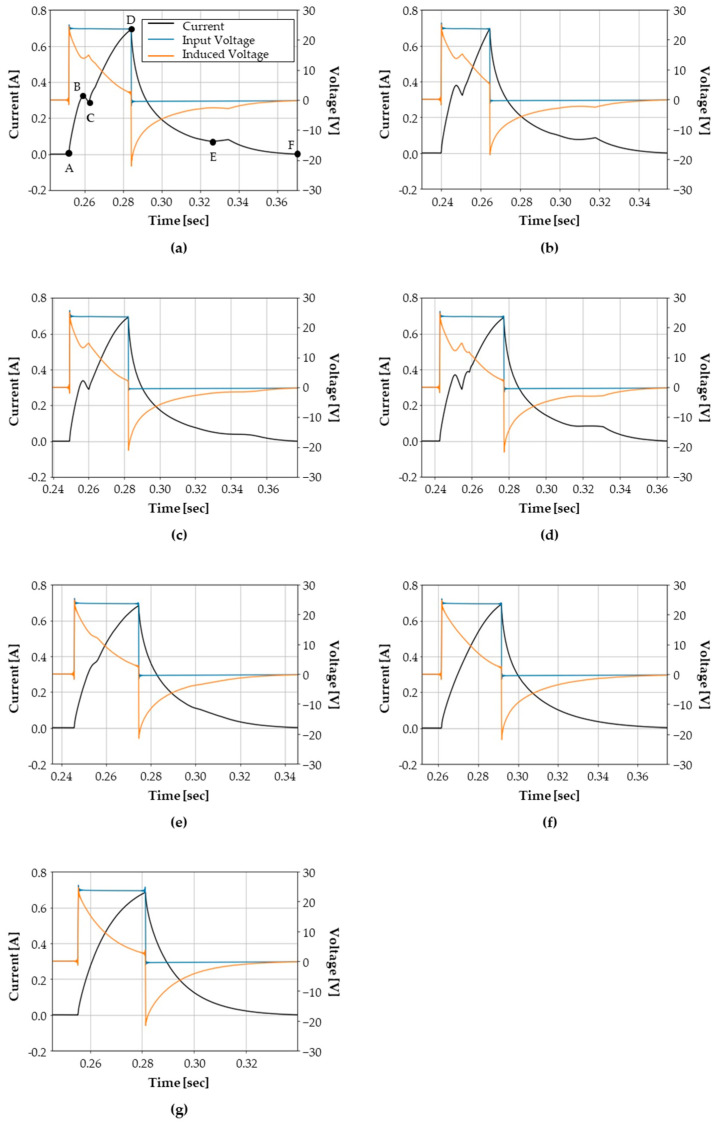
Measured valve data for each health state: (**a**) normal, (**b**) abnormal coil turn, (**c**) abnormal spring stiffness, (**d**) missing spacer, (**e**) stuck spool, (**f**) plunger adhesion on the top, and (**g**) plunger adhesion on the bottom.

For a normal hydraulic solenoid valve, which is labeled as Class-1, the voltage and current signals are presented in Figure 11a. When the power switch is turned on, voltage is applied to the coil, and the current increases from point A to point B in Figure 11a according to the characteristics of the first-order response of the LR circuit. Once the current reaches a certain level (point B), the plunger starts to move, which generates counter-electromotive force. At this point, the current decreases due to the counter-electromotive force, which is shown as point B to point C in Figure 11a. After that, the plunger moves until it reaches its maximum position, which makes the current increase again; this is presented as point C to point D. At the moment when the current reaches its maximum value, the valve controller turns off the power switch at point D.

From this point, the current is supplied to the solenoid coil through the freewheeling diode, and a negative voltage is generated across the coil terminals due to the voltage drop across the diode. When the current decreases to a certain extent as from point D to point E, the plunger returns to its original position due to the return spring. At this moment, the current increases temporarily due to the movement of the plunger, and, after that, the current decreases to zero, which is from point E to point F. Figure 11 presents the measured data from seven different samples, as shown in Table 1. Note that the measurement time for the valve diagnosis is within 0.15 s.

**Figure 12:**
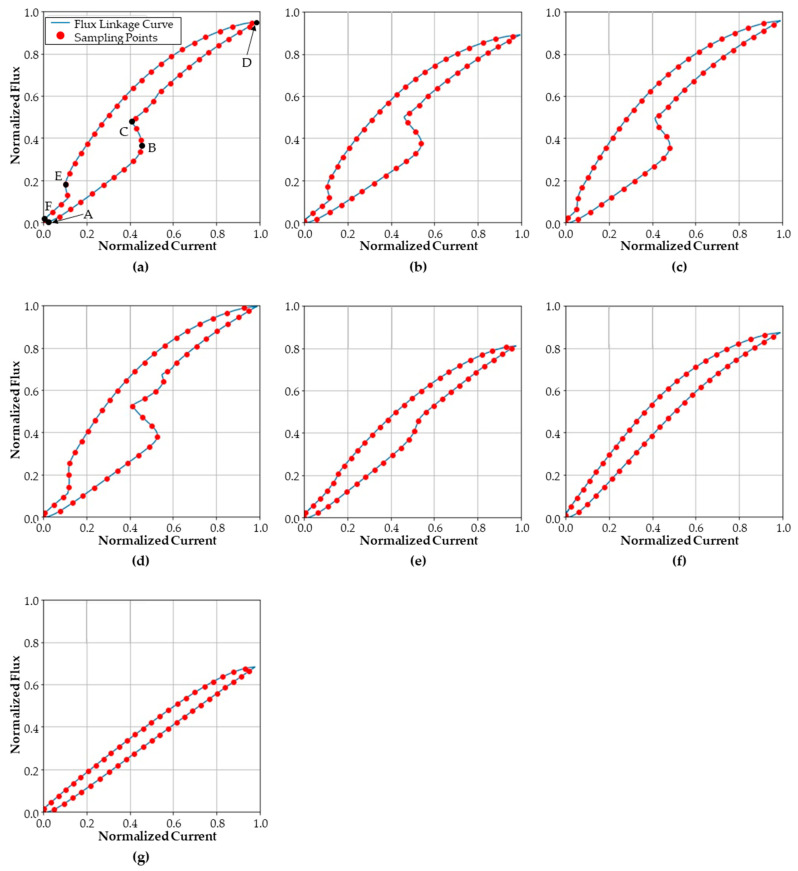
Current–flux linkage curve for each health state: (**a**) normal, (**b**) abnormal coil turn, (**c**) abnormal spring stiffness, (**d**) missing spacer, (**e**) stuck spool, (**f**) plunger adhesion on the top, and (**g**) plunger adhesion on the bottom.

Using the measured data, an x–y plot of current and the rate of change of flux linkage is presented in Figure 12. Each figure corresponds to each health state in Figure 11. The red dots indicate 50 sampled points evenly spaced in the normalized current–voltage plane, as described in Section 3.1. Here, the difference of flux linkage is obtained by integrating the induced voltage according to Equation (2). Both the current and the flux linkage are normalized using Equations (5) and (6), respectively. Note that the maximum current $\left(i_{max}\right)$ is set to 700 mA, and the maximum flux linkage $\left(\Psi_{max}\right)$ is set to 0.35 Wb.

The curve of each health state is displayed in Figure 12. For the normal state, the counter electromotive force creates a vertex point C on the curve. For an abnormal coil turn case, a larger current is required to achieve equal flux, as compared to the normal state. For the abnormal return spring case, a slightly moderate vertex between points E and F can be observed, as compared to that of normal case. The missing spacer valve causes unstable movement of the plunger; as a result, the curve shows a stronger zigzag shape between points C and D. Moreover, among the seven health states, the highest flux is reached in this condition. In the case of the stuck spool, the deformation BC of the curve due to the plunger movement takes a smoother curve than that of the normal case. The adhesion of the plunger at the top and bottom presents a relatively simple shape with only a vertex at point D. The curves of last two health states present a similar shape; however, they have different maximum flux values.

**Figure 13:**
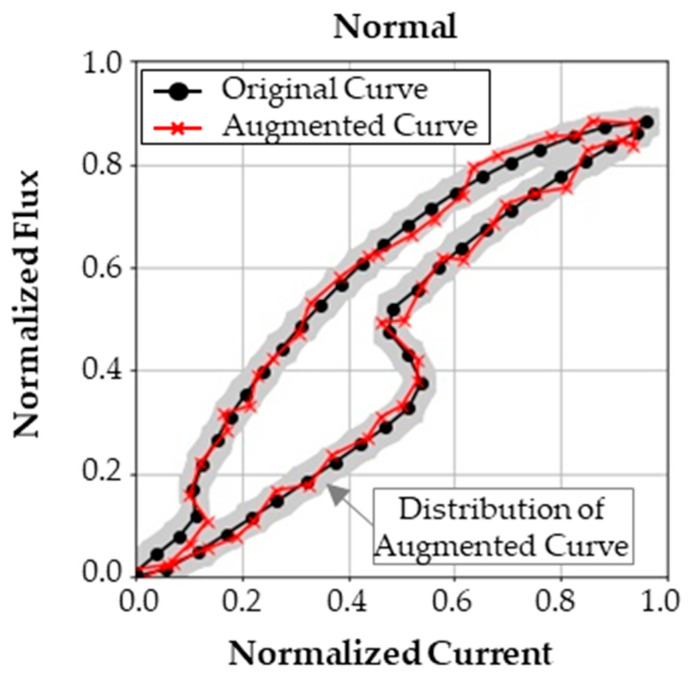
Current–flux linkage curve and its augmented sample curve.

The current–flux linkage curve data in Figure 12 are resampled to forty-eight-by-two-sized vector data. To extract the latent feature vector from the curve data, the curve data set was augmented with white noise, as described in Section 3.1.3. Noise from a uniform distribution of ±2.5% magnitude was added to the normalized current and flux linkage sample points. Figure 13 displays an example of the original curve data and the augmented curve data.

**Figure A1:**
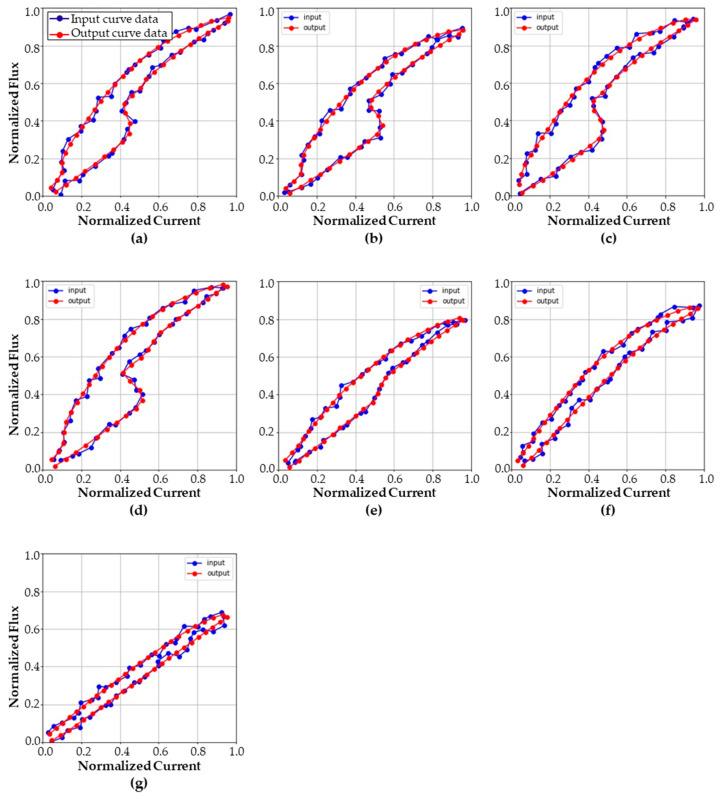
Reconstruction of current–flux curve with the autoencoder for noisy data: (**a**) normal, (**b**) abnormal coil turn, (**c**) abnormal spring stiffness, (**d**) missing spacer, (**e**) stuck spool, (**f**) plunger adhesion on the top, and (**g**) plunger adhesion on the bottom.

For each class, the test was repeated five times, which produced five curves for each class. The noise was added to each curve, and each curve was augmented to 1000 curves. In the end, 5000 curve data for each class were obtained. Examples of augmented curves are presented in Figure A1.

### 5.2. Results of Feature Extraction

From the augmented curve data set, latent feature vectors were extracted using a convolutional autoencoder (CAE), as described in Section 3.2. The bottleneck latent layer was set to 12 to learn latent features of the curve data. Among the 5000 augmented curve data of each class, 90% were randomly selected from each individual class for training the CAE, and the remaining 10% were used for verification. The training of the CAE was conducted for 200 epochs with a batch size of 100, where the validation split was set to 10% of the training data. The learning time of CAE took 401 s on a laptop equipped with a CPU with a clock speed of 2.4 GHz and an NVIDIA GeForce RTX 2080 GPU.

**Figure 14:**
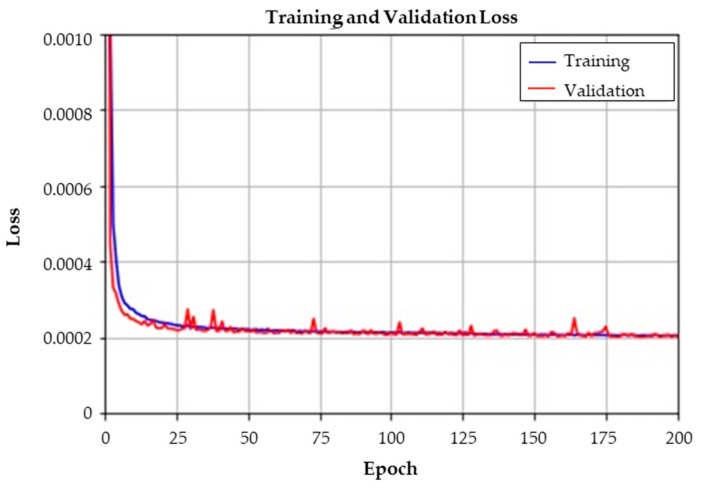
Training and validation loss of the convolutional autoencoder.

**Figure 15:**
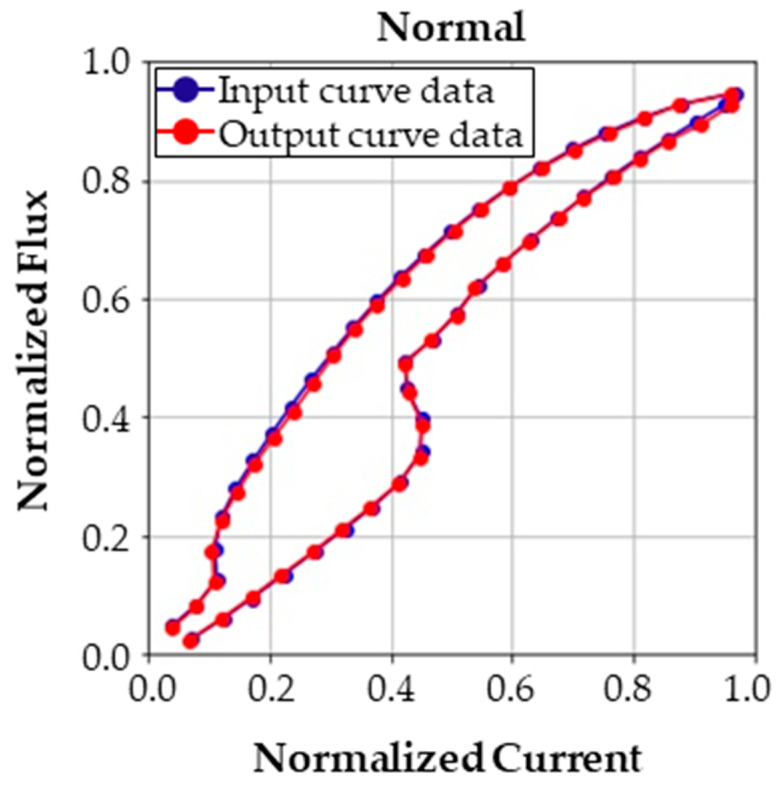
Input sample curve data and the corresponding output curve data from the trained autoencoder for normal case. Rest of the cases are illustrated in Figure A2.

Figure 14 represents the mean average error (MAE) loss between the input and the output of the CAE. As the CAE is trained, MAEs of both the training set and validation set decrease. In other words, the trained CAE can thoroughly restore the input data. An example of the restored output of each class is presented in Figure 15. The blue lines denote input curve data, and the red lines denote the restored curve data from the decoder. The results show that the input data are almost perfectly restored; thus, we can conclude that the CAE has learned features of the curves. Although the dimension of the latent variable was set to twelve, the CAE restored the input quite well.

### 5.3. Results of Fine-Tuning the Feature Extractor

**Figure 16:**
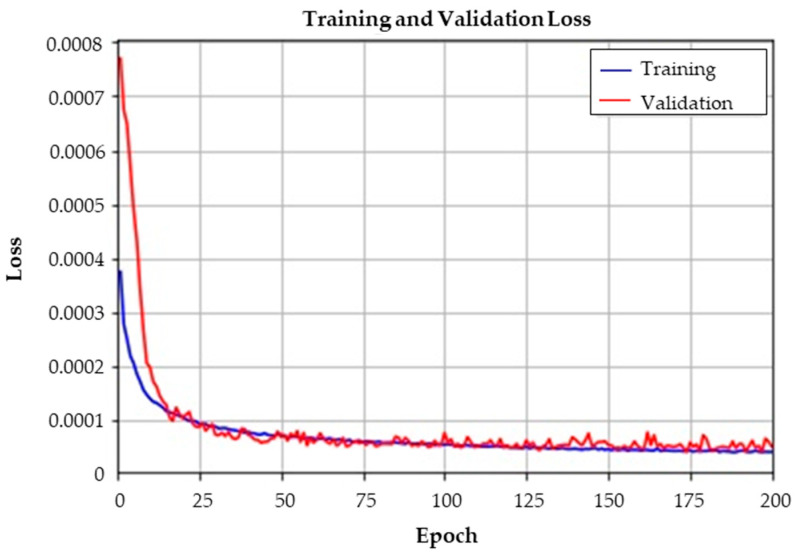
Training and validation loss of the convolutional autoencoder fine-tuning.

The latent feature vector of dimension size 12 was acquired by training the convolutional autoencoder (CAE). From the extracted latent feature vectors, the center point of each class in the 12-dimension space was calculated as described in Equation (8), where the feature vector is calculated for all training data (i.e., *N* = 4500). As densely distributed data of the same class are preferable, the CAE is trained again with the loss function defined in Equation (10). The fine-tuning process tries to extract features that are more densely distributed for each class, by reducing the distance between the sample and the center point. The results of fine-tuning are shown in Figure 16. Note that the loss in Figure 16 is calculated by Equation (10), while the loss in Figure 14 is calculated by Equation (7). All hyperparameters other than the loss term were set identical to those of the CAE, and the learning process took 201 s. Since the fine-tuning starts with the previously trained CAE, the initial loss for training and validation is relatively low. Also, the validation loss as well as the training loss decreases steadily, which indicates that the autoencoder weights are updated to present more densely distributed data of each class in the latent feature vector space.

**Figure 17:**
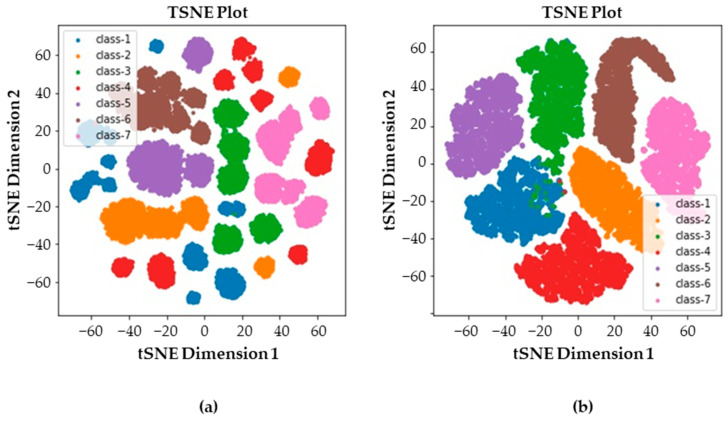
t-SNE plots of latent feature vectors: (**a**) before fine-tuning and (**b**) after fine-tuning.

**Figure 18:**
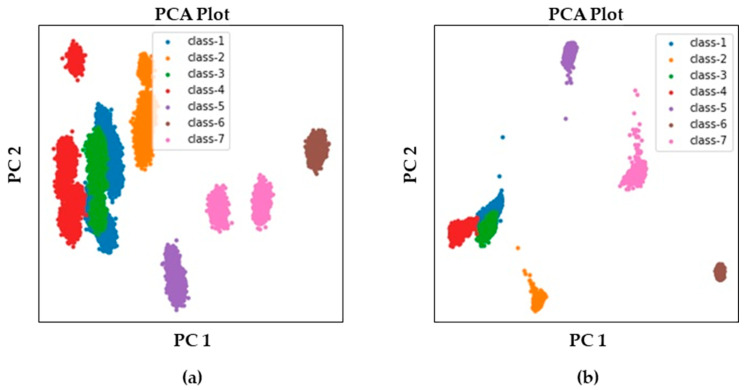
Principal component distributions of latent feature vectors: (**a**) before fine-tuning and (**b**) after fine-tuning.

**Figure 19:**
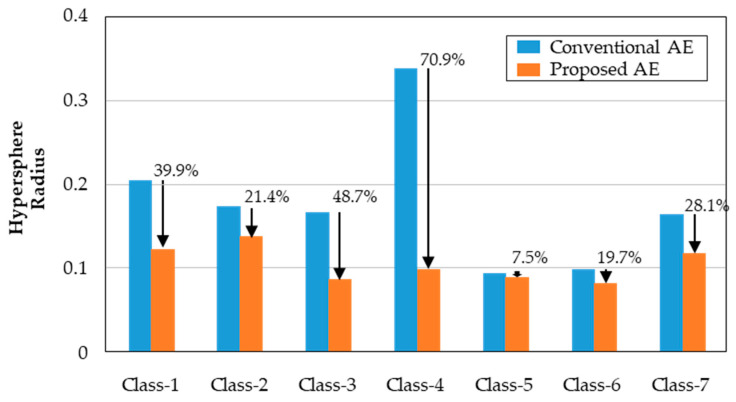
Radius of each hypersphere produced by a conventional autoencoder and the proposed autoencoder.

The effect of the fine-tuning can be easily seen by the t-distributed stochastic neighbor embedding (t-SNE) plots as shown in Figure 17. Figure 17a presents a t-SNE plot of the latent feature vector before fine-tuning, which is extracted from the conventional autoencoder. In contrast, Figure 17b presents a t-SNE plot of the latent feature vector after fine-tuning, which is the result of the proposed method. Each color represents each class of data in the Figure. The feature vectors developed by the proposed method form more dense clusters than those of the feature vectors found by the conventional autoencoder. A similar result can be derived from principal component analysis (PCA). The distributions of two-principal components are shown in Figure 18. Similar to the t-SNE plots, the proposed fine-tuned latent feature vector displays more clustered distributions than that of conventional latent feature vectors. Although Figure 17 and Figure 18 indicate the results qualitatively, Figure 19 compares the actual hypersphere radius of each health state. As the fine-tuning used the loss function to reduce the distance between the samples and the center of the corresponding class, the results show that fine-tuning actually reduced the distance and the radius of the hypersphere. The reduction in the hypersphere was the largest at class-4, which was 70.9%. Other radiuses of hyperspheres decreased as well, averaging around 33.7%. Eventually, the fine-tuned latent feature vector improved the fault-classification accuracy.

### 5.4. Classification Results

The latent feature vectors from the fine-tuned convolutional autoencoder (CAE) were used for the classification. The classification was performed on the latent feature vector space of 12 dimensions. Each hypersphere indicates a single class, which is a type of health state of a hydraulic solenoid valve. If a verification sample lies in an overlapped section of two or more hyperspheres, the verification sample is classified as the class represented by the region with the shortest distance between the center of the hypersphere and the sample.

**Figure 20:**
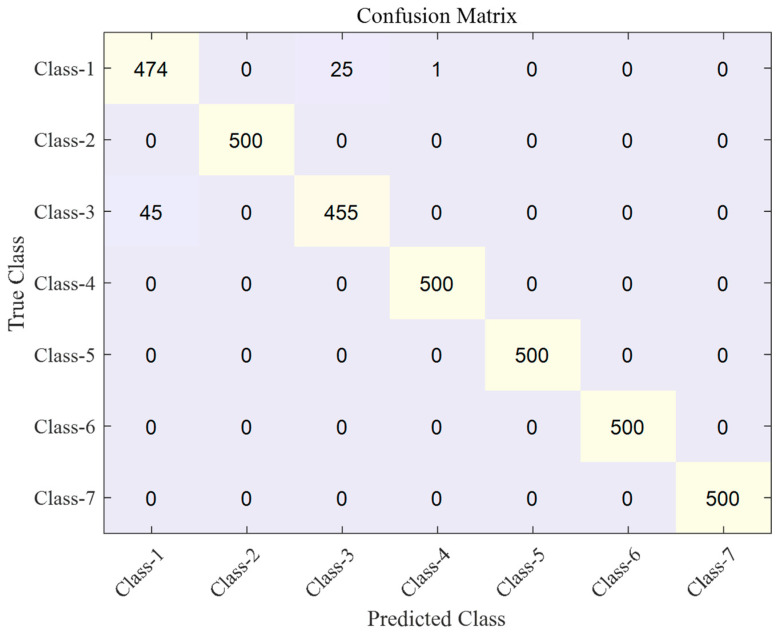
Confusion matrix of the hydraulic solenoid valve classification derived by a conventional autoencoder.

**Figure 21:**
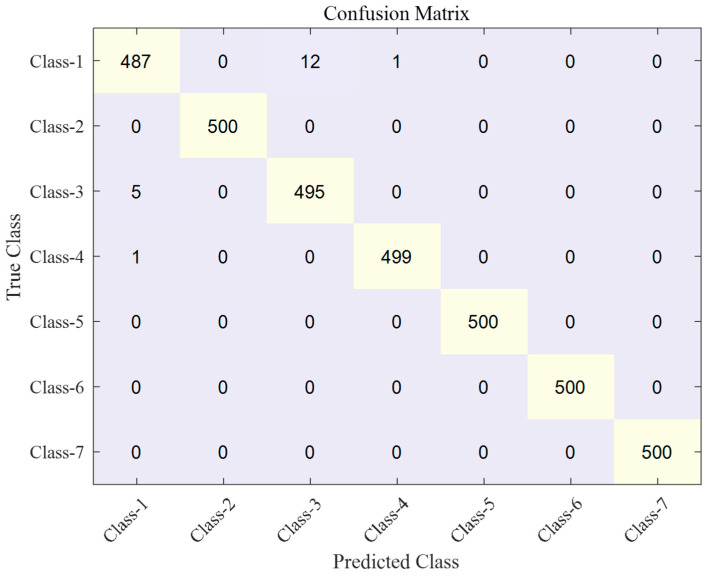
Confusion matrix of the hydraulic solenoid valve classification developed by the proposed autoencoder.

The classification results are shown as a confusion matrix in Figure 20 and Figure 21. Figure 20 presents the results of the conventional autoencoder without a fine-tuning stage, whereas Figure 21 presents the results of the proposed autoencoder with a fine-tuning stage. The overall classification accuracies of the two methods are 97.9% and 99.5%, respectively. By reducing the distance metric, the latent features are densely clustered, which leads to the increase in the classification performance.

Specifically, the misclassified samples are mostly due to samples from class-1 and class-3. As the curves of the two classes, displayed in Figure 12a,c, have a similar shape, the two classes are misclassified as each other. The distribution of the latent feature vector also shows that the two-classes are close to each other; this is shown in Figure 17 and Figure 18. The two clusters of class-1 and class-3 are partly overlapped, whereas other clusters are not overlapped at all. Thus, the classification accuracy of the two classes is 92.9% for the conventional autoencoder method. As the features are densely clustered by the proposed fine-tuning stage, the classification accuracy increases to 98.2%.

**Figure 22:**
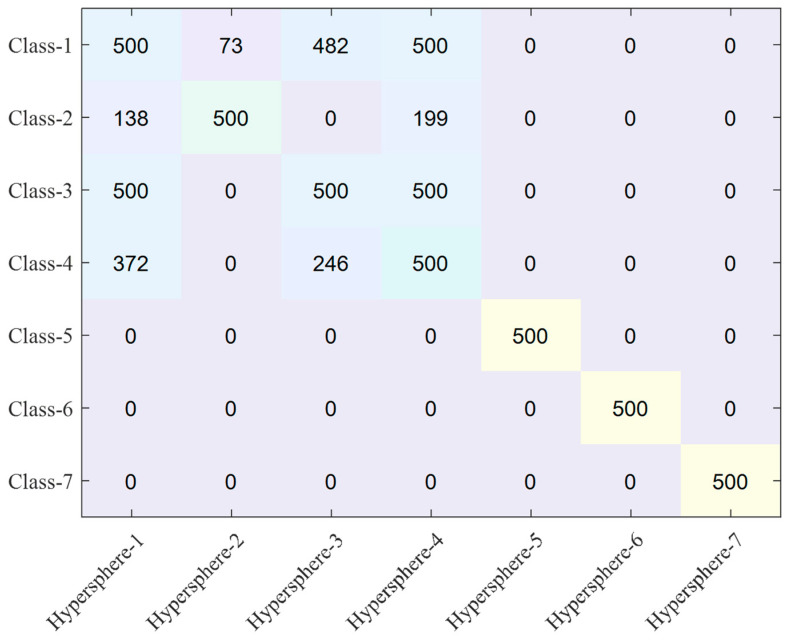
Number of samples included in each hypersphere for the conventional autoencoder.

**Figure 23:**
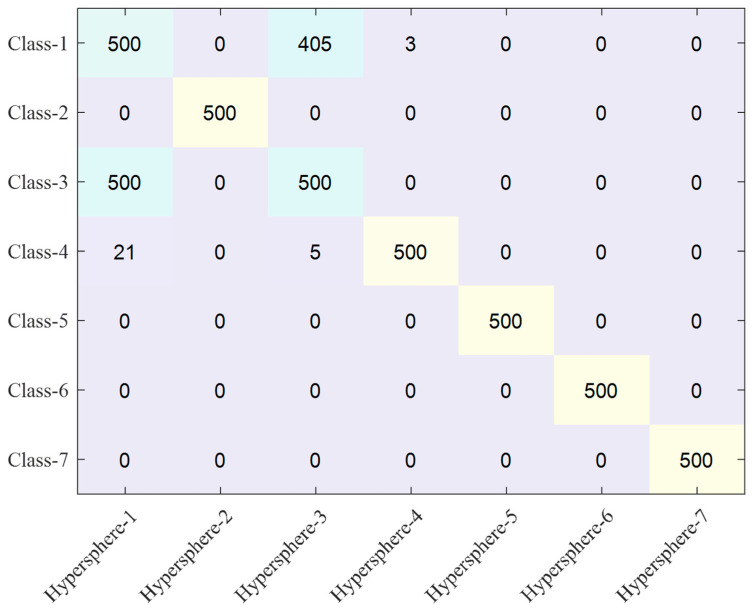
Number of samples included in each hypersphere for the proposed autoencoder.

**Figure A3:**
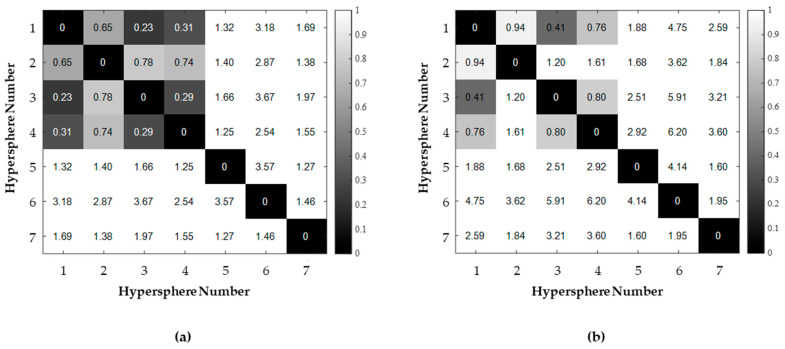
Degree of overlap between two hyperspheres: (**a**) a conventional autoencoder and (**b**) the proposed autoencoder.

The actual number of samples included in each hypersphere is presented in Figure 22 and Figure 23. Based on the conventional autoencoder method, hypersphere-1 defining class-1 includes not only data from class-1 but also 138 samples from class-2, 500 samples from class-3, and 372 samples from class-4. This indicates that a significant overlap region exists among hyperspheres. Naturally, hyperspheres-2, -3, and -4 contain samples from other classes. However, after fine-tuning, such overlapping regions between the hyperspheres significantly decrease, as shown in Figure 17 and Figure 18. The fine-tuning clearly reduces the overlapped regions, which leads to improved classification accuracy, as shown in Figure 22. Although hyperspheres-1 and -3 are still overlapped significantly, all other hyperspheres include their own accurate class samples. The degree of overlap is shown in Figure A3.

## 6. Conclusions

This study proposed a convolutional autoencoder based fault-diagnosis method for hydraulic solenoid valves (HSVs). Specifically, current–flux linkage data were derived from the valve and were resampled at equal intervals to capture geometry features. In addition, white noise was added to the original signal to augment the data set. Then, latent feature vectors representing the shape of the current–flux linkage curve were extracted through a one-dimensional convolutional autoencoder. The latent feature vectors were then classified using hyperspheres capable of classifying various fault types. As the autoencoder was fine-tuned by minimizing the distance between the samples and the center of the hypersphere, the prediction accuracy increased. In addition, the hypersphere-based classification scheme was developed to consider an unknown fault mode in the validation stage.

To validate the proposed method, an experimental testbed was set up. A normal state and six types of faults were tested; current and voltage signals were acquired for each state. A total of seven classes were trained and validated. The results show that the classification accuracy of the proposed approach was higher than the prediction accuracy of the conventional autoencoder approach. The distribution of the latent feature vector indicates that the fine-tuning reduces the overlapped region and predicts the samples more accurately.

The suggested method can diagnose the valve in real time using the current and voltage signals of the valve coil. Also, the suggested method can be implemented with a software update to an existing HSV controller, without any additional sensors. In comparison to previous methods, the approach developed in this paper uses an autoencoder based approach, allowing accurate fault diagnosis of HSVs and identification of unknown faults. As the method is light and accurate, the fault-diagnosis strategy can be applied to the assembly line of hydraulic valves. In addition, it can also be applied effectively to valves installed on machines.

It should be noted that the proposed fault diagnosis method focuses only on the faults on the internal components of the valve, such as abnormal coil turns, abnormal spring stiffness, spacer missing, spool stuck, and plunger adhesion. In contrast, the proposed method does not address external faults that appear on the external components of the valve, such as seal degradation, blockages of the port, surface cracks, etc. Also, the proposed method aims to diagnose the valve malfunctions before the start of the hydraulic system operation, and it is not eligible for the immediate detection of faults during operation.

In future research, the learning algorithm will be supplemented to minimize the overlap between hyperspheres to improve the classification accuracy further. Furthermore, the newly developed autoencoder model will be optimized to implement in an actual valve controller through embedded software. In addition, further research into IoT-based operation will be conducted, so that data collection, data management, model training, and distribution via over-the-air (OTA) will be possible.

## Figures and Tables

**Table 1:** Description of test specimens.

Class	Fault Mode	Description
1	Normal	Coil turns: 1700 Coil diameter: 0.3 mm Coil resistance: 30 Ohm Spring stiffness: 0.06 kgf/mm Initial compression: 2.5 mm
2	Abnormal coil turn	Coil turns: 1650 Coil diameter: 0.32 mm Coil Resistance: 26 Ohm
3	Abnormal spring stiffness	Spring stiffness: 0.07 kgf/mm Initial compression: 1.0 mm
4	Spacer missing	Omission of the part
5	Spool stuck	Increased friction coefficient by applying foreign substances to the surface of the spool
6	Plunger adhesion on top	Adhesion between plunger and valve housing due to contaminants
7	Plunger adhesion on bottom	

**Figure A2:**
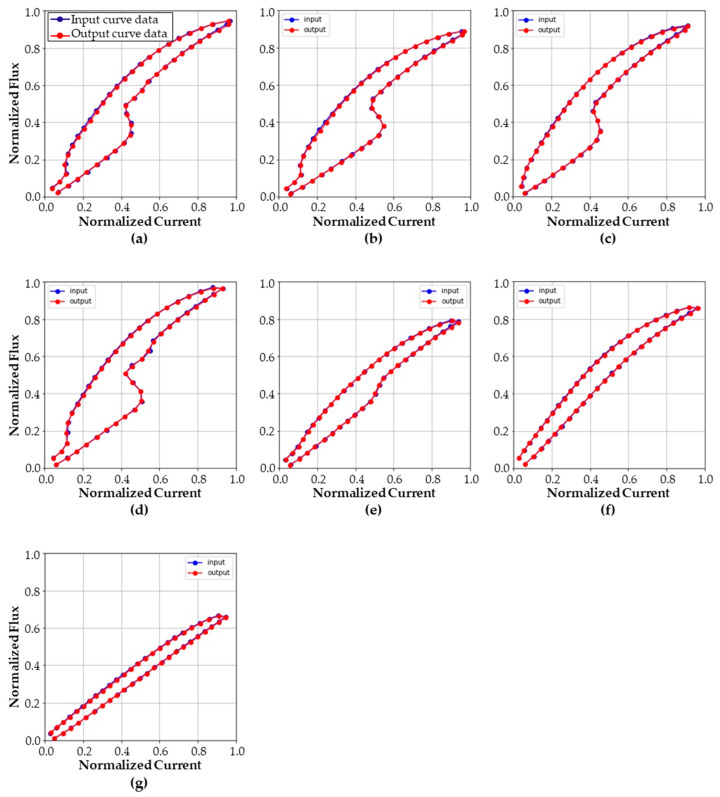
Input sample curve data and the corresponding output curve data from the trained autoencoder: (**a**) normal, (**b**) abnormal coil turn, (**c**) abnormal spring stiffness, (**d**) missing spacer, (**e**) stuck spool, (**f**) plunger adhesion on the top, and (**g**) plunger adhesion on the bottom.

## Data Availability

The data are not publicly available due to security issues.

## Appendix A

Degree of overlap of two hyperspheres, $O\left(c_{i},R_{i}\right)$ and $O\left(c_{j},R_{j}\right)$, are defined as the following equation.

(A1) $$d_{i,j}=\frac{\left\|c_{i}-c_{j}\right\|}{R_{i}+R_{j}}$$

$c_{i}$ and $R_{i}$ denote the center and the radius of $i^{th}$ hypersphere, respectively. If $d_{i,j}$ is larger than 1, two hyperspheres are overlapped. Conversely, if $d_{i,j}$ is smaller than 1, two hyperspheres are not overlapped. Figure A3 presents the degree of overlap for all combinations of two hyperspheres. The fine-tuning autoencoder increases the degree, which indicates that the overlapped region is decreased.
